# Postpartum Hemorrhage in Women with Von Willebrand Disease – A Retrospective Observational Study

**DOI:** 10.1371/journal.pone.0164683

**Published:** 2016-10-25

**Authors:** Igor Govorov, Signe Löfgren, Roza Chaireti, Margareta Holmström, Katarina Bremme, Miriam Mints

**Affiliations:** 1 Department of Women’s and Children’s Health, Karolinska Institutet, Karolinska University Hospital, Solna, 171 76, Stockholm, Sweden; 2 Department of Molecular Medicine and Surgery, Karolinska Institutet, Solna, 171 76, Stockholm, Sweden; 3 Department of Medicine, Karolinska University Hospital Solna, 171 76, Stockholm, Sweden; Stellenbosch University, SOUTH AFRICA

## Abstract

**Introduction:**

von Willebrand disease (VWD) is a hereditary bleeding disorder, caused by a deficiency in the levels and/or function of von Willebrand factor (VWF). Women with VWD appear to be at increased risk of experiencing postpartum hemorrhage (PPH), though the levels of VWF increase during pregnancy. There is limited knowledge of how PPH is associated with the subtype of VWD, plasma levels of other coagulations factors than VWF and given hemostatic treatment.

**Aims:**

The aims were to investigate the incidence of PPH in women with VWD and to analyse the correlation between PPH and: (1) type of VWD, (2) laboratory monitoring of VWF and FVIII and (3) hemostatic drug treatment.

**Methods:**

This was a retrospective observational study. The study participants (n = 34) were recruited from the Coagulation Unit, Karolinska University hospital. Fifty-nine deliveries, which occurred in 14 different obstetrics units (years 1995–2012) were included in the study.

**Results:**

The incidence of primary PPH was 44%, severe primary PPH 20% and secondary PPH 12%. VWD type 3 was associated with a higher risk of experiencing severe primary PPH compared to other subtypes. FVIII:C in pregnancy was inversely correlated to blood loss during delivery. There was a significantly higher incidence of secondary PPH when the VWD diagnosis was unknown at time of delivery.

**Conclusions:**

The women with VWD are at higher risk of PPH, especially those with type 3 VWD or when diagnosis is unknown prior to delivery. Identification of pregnant women with undiagnosed VWD may be of importance in order to prevent PPH.

## Introduction

Von Willebrand disease (VWD) is a hereditary bleeding disorder first described in 1926 by Erik von Willebrand [1]. VWD is caused by deficiency of von Willebrand factor (VWF). VWF is a blood glycoprotein, which have dual function: it facilitates platelet adhesion and aggregation and also serves as the carrier of coagulation factor 8 (FVIII). The most common is type 1(more than 80% of cases)—partial quantitate deficiency of VWF, type 3 is the most rare one (less than 1% of cases) and corresponds to almost total absense of VWF. Type 2 accounts for 15–20% of cases and is further subdivided into 4 subtypes (2A, 2B, 2M and 2N), according to different impairments in form and/or function of VWF [2–4].

To date, the prevalence of VWD varies depending on calculation: epidemiological studies report it to be approximately 0.5–1% [5, 6], making VWD the most common inherited bleeding disorder, whereas the prevalence based on the referral rate of VWD patients to specialized coagulation centres is reported to be 0.002–0.01% [7]. In the Nordic region, the prevalence of VWD is approximately 0.008% based on referral rate [8].

Despite the fact that both males and females are affected by VWD, women face additional specific hemostatic challenges during menstruation and childbirth [9]. Excessive bleeding after delivery, called postpartum hemorrhage (PPH) is the leading cause of maternal morbidity and mortality globally, especially in the developing world [10]. PPH can be divided into primary and secondary PPH. Primary PPH is traditionally defined as blood loss of 500 ml or more and severe PPH as 1000 ml or more within 24 hours of delivery [10, 11]. Secondary PPH is defined as any abnormal bleeding 24 hours to six weeks postpartum, regardless of volume. In high-resource countries where uterotonic drugs are used routinely, the reported incidence of primary PPH, severe primary PPH and secondary PPH is respectively 5–19%[12–14], 4–10% [13, 15, 16] and 1–3% [17].

Studies have shown a significantly increased incidence of PPH in women with VWD compared to healthy controls [18–24], despite the fact that FVIII and VWF increase during pregnancy [25]. Experts have suggested that if plasma FVIII is at least 30–40% of normal levels, then bleeding after delivery is unlikely [26]. The majority of these studies are based on patient recall and do not take medical records of estimated blood loss or administration of hemostatic drugs into account.

In Sweden there are currently no national guidelines for the obstetric management of the VWD patient. Thus, the clinical approach may vary between different regions and hospitals. Also, there is limited knowledge of how PPH correlates to the subtype of VWD, levels of VWF and FVIII during pregnancy and hemostatic drug treatment before and after delivery. A better understanding of these factors could help create clinical guidelines for women with VWD during pregnancy, delivery and postpartum, which may help decrease the risk of PPH and the associated complications in women with VWD.

The aims of this study were to investigate the incidence of PPH in women with VWD and to analyse the associations between PPH and subtype of VWD, levels of VWF and FVIII in pregnancy and hemostatic drug treatment.

## Materials and Methods

### Patients and medical records

This study design is retrospective and observational.

**Fig 1:**
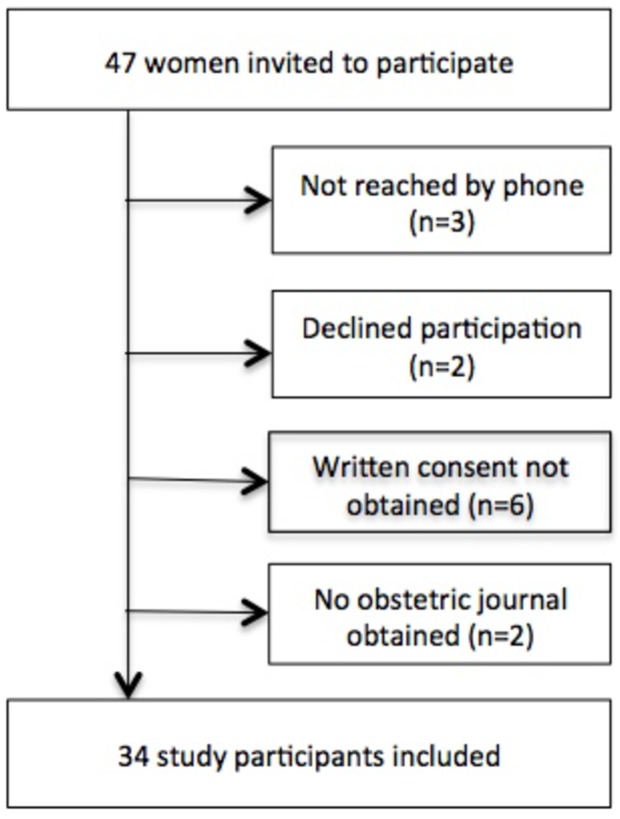
Flowchart for inclusion process.

The study group was recruited from a local registry at the Coagulation Unit, Karolinska University Hospital, which contains comprehensive demographic and clinical data, which is considered as personal information. However, for scientific purposes data was then de-identified by the authors and handled in this manner. The local registry is a database that accumulates clinical data about patients with coagulation disorders from all across Sweden both for clinical and scientific purposes. As soon as a patient is diagnosed with bleeding disorder, he/she is being included in the registry and due to existence of national database in Sweden his/her clinical history can be tracked back in order to collect necessary clinical information, *e*.*g*. delivery nuances. During current study database was sorted in agreement with chosen inclusion criteria. The inclusion criteria were: VWD diagnosis (all subtypes), female gender, age 18–50 years and an obstetric history of at least one delivery. The upper age limit was chosen in order to focus on deliveries during the last 20 years and also due to foreseen difficulties in obtaining older medical records. All women who met the inclusion criteria (n = 47) were identified from the registry and invited to participate, 34 of them were included in the final cohort (Fig 1).

In total, 34 women and 59 deliveries were included in the study. Maternal age ranged from 19 to 42 years (median 32).

For each delivery, information was obtained regarding maternal age, the hospital for delivery, mode of delivery and complications associated with delivery. Blood loss assessment was performed by weighing clots and soaked materials.

The diagnosis of vWD was made according to well-recognized criteria: bleeding episodes, family history and low levels of vWF. Different types of vWD were distinguished using RCoF activity and vWF:Ag tests and its ratio.

Data was also obtained regarding treatment with tranexamic acid (TXA), desmopressin (DDAVP^®^) and clotting factor concentrate (CFC) prior to delivery and in the postpartum period (type of treatment, dose prior to delivery, duration of treatment and total dose).

### Laboratory measurement of von Willebrand factor

VWF activity was measured using two methods (VWF:RCo and VWF:GpIb) depending on which of them was used at the time of the measurement at the Karolinska University Laboratory.

VWF:RCo (0.08–0.86 kIU/L) measures ristocetin cofactor activity and is based on the ability of antibiotic ristocetin to induce binding of the VWF to the platelet receptor glycoprotein Ib (GpIb) and thus lead to agglutination [27]. Platelet-poor plasma from venous blood is combined with a reagent containing ristocetin and formalin-fixed platelets. The rate of agglutination is analysed by photometry as an increase of absorbance [28].

VWF:GpIb (0.08–0.86 kIU/L), a ristocetin independent method, is currently used to measure VWF activity at the Karolinska University Laboratory [29]. According to this method, the patient’s plasma is mixed with a reagent containing recombinant GpIb receptors to which VWF may bind without the presence of ristocetin. As for VWF:RCo, the rate of agglutination is measured by absorbance. The units and reference range for VWF:RCo and VWF:GpIb are identical, making it possible to compare results between the two methods [28, 29]

FVIII activity (0.06–2.10 kIU/L) is analysed using a well established enzymatic method [30].

In current study, we used reference ranges for third trimester, accepted at Coagulation Unit Lab.

### Ethical permission

The participants were informed about the purpose of the study and that participation was voluntary. Written informed consent was obtained from all participants and data was made anonymous directly after collection. A previous ethical permission, granted to the authors (Dr.Miriam Mints), was extended (registration number 2007/1373-31/4) to include the present study. The current study was reviewed by the Karolinska Institute Ethical Board before the extension on registration number 2007/1373-31/4 was granted.

### Statistical methods

The study group was arranged in subgroups according to the type of VWD, the laboratory tests performed and the type of hemostatic drug treatment administered. Due to the small sample sizes, Fisher’s exact test was used to compare the incidence of PPH between the groups.

The Kruskal-Wallis test was used to compare blood loss(ml) between three subtypes of VWD. The correlation between blood loss and plasma levels of VWF and FVIII in third trimester pregnancy appeared to be non-linear and was assessed using Spearman’s correlation test. The same test was used to analyse the correlation between blood loss and prophylactic hemostatic drug treatment (dose prior to delivery).

Mann-Whitney U test was used to compare the treatment (duration of treatment and total dose) between the groups.

Statistical significance was set at p< 0.05. Statistical analyses were performed using IBM SPSS statistics software version 21.

## Results

### Study group

Thirty-four women who gave birth to 61 children were included in the study (Table 1). Fifty-nine deliveries at 14 different obstetric clinics in Sweden during 1995–2012 were included. In 28 women (43 deliveries), the diagnosis of VWD was known before delivery. In 11 women (16 deliveries), the diagnosis of VWD was made following delivery. Five women were included in both groups since the diagnosis of VWD was determined in between two pregnancies.

**Table 1:** Study participants and deliveries by subtype of VWD.

		Type 1	Type 2	Type 3	Unknown	All
Participants		21	9	3	1	34
Deliveries (%)		39	14	4	2	59
Mode of delivery (%)	Vaginal	29 (74.4)	9 (64.3)	1(25)	2 (100)	41 (69.5)
	Instrumental vaginal	4 (10.3)	2 (14.3)	1 (25)	-	7 (11.9)
	Caesarean	6 (15.4)	3 (21.4)	2 (50)	-	11 (18.7)
Induced labour (%)		7 (17.9)	5 (35.7)	1 (25)	-	13 (22.0)
Maternal age (%)	≤23	4 (10.3)	-	-	-	4 (6.8)
	24–30	13 (33.3)	3 (21.4)	1 (25)	1 (50)	18 (30.5)
	31–37	18 (46.2)	8 (57.1)	3 (75)	1 (50)	30 (50.8)
	≥38	4 (10.3)	3 (21.4)	-	-	7 (11.9)
Parity (%)	1	20 (51.3)	7 (50)	3 (75)	1 (50)	31 (52.5)
	2	13 (33.3)	4 (28.6)	1 (25)	1 (50)	19 (32.2)
	3 or more	6 (15.4)	3 (21.4)	-	-	9 (15.2)
Birth weight (%)	<2499	2 (5.1)	1 (7.1)	-	-	3 (5.1)
	2500–3999	31 (79.5)	11 (78.6)	4 (100)	2 (100)	48 (81.4)
	>4000	6 (15.4)	2 (14.3)	-	-	8 (13.6)
Gestational age (%)	<36	2 (5.1)	-	1 (25)	-	3 (5.1)
	36–41	30 (76.9)	12 (85.7)	3 (75)	2 (100)	47 (79.7)
	>41	7 (17.9)	2 (14.3)	-	-	9 (15.2)
Obstetric unit in close connection with a coagulation unit (%)		21 (53.8)	8 (57.2)	3 (75)	-	32 (54.3)
Plasma levels checked in pregnancy (%)		26 (66.7)	13 (92.9)	4 (100)	-	43 (72.9)
Median VWF:RCo/VWF:GpIb, kIU/L (range)		0.55(0.08–0.86)	0.21(0.08–0.68)	0.08(0.08–0.24)	-	0.25(0.08–0.86)
Median FVIII:C,kIU/L (range)		1.07(0.32–2.10)	0.84(0.63–1.86)	0.70(0.06–1.17)	-	0.94(0.06–2.10)
No haemostatic treatment (%)		13 (33.3)	1 (7.1)	-	2 (100)	16 (27.1)
TXA (%)		7 (17.9)	2 (14.3)	-	-	9 (15.3)
TXA and DDAVP (%)		11 (28.2)	1 (7.1)	-	-	12 (20.3)
TXA and CFC (%)		8 (20.5)	10 (71.4)	4 (100)	-	22 (37.3)

Characteristics regarding mode of delivery, maternal age, parity, birth weight and gestational age, place of delivery, levels of VWF and FVIII in late pregnancy and the haemostatic drug treatment administered during and after delivery.

Abbreviations: VWD = von Willebrand disease; VWF:RCo = von Willebrand factor activity; FVIII:C = Factor VIII activity; TXA = tranexamic acid; DDAVP = desmopressin; CFC = clotting factor concentrate containing VWF and FVIII.

In all women where the diagnosis of VWD was known before delivery, the levels of VWF and FVIII were analysed in the third trimester of pregnancy (gestation week 26–37).

The levels of VWF activity in plasma were subnormal in all pregnancies in women with VWD type 3 (n = 4), in 92,3% of pregnancies in VWD type 2 (12/13) and in 38,5% of pregnancies in VWD type 1 (10/26). The plasma levels of FVIII were subnormal in 50,0% of pregnancies in VWD type 3 (2/4), no pregnancies in VWD type 2 (0/13) and in 19,2% of pregnancies in VWD type 1 (5/26).

### Incidence of PPH

The incidence of primary PPH was 44% (n = 26), severe primary PPH—20% (n = 12), secondary PPH—12% (n = 7). Primary PPH occurred in 37% of vaginal deliveries, 57% of instrumental vaginal deliveries and 64% of caesarean sections. Severe primary PPH complicated 17% of vaginal deliveries, 43% of instrumental vaginal deliveries and 18% of caesarean sections.

**Fig 2:**
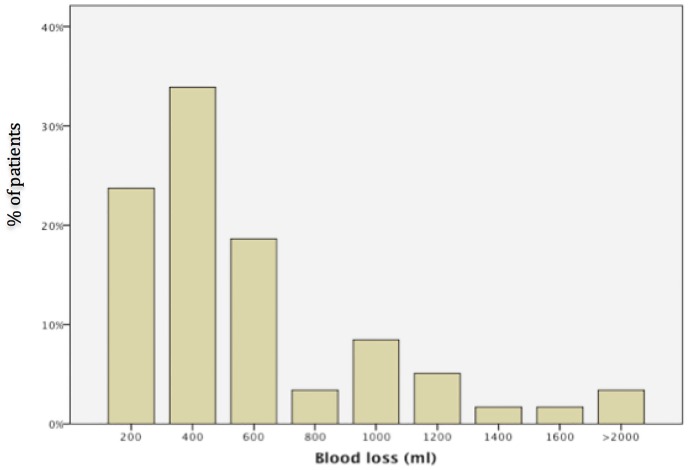
Distribution of blood loss.

Three women needed blood transfusions in the postpartum period (5%) and vaginal hematoma occurred in three deliveries (5%). In one delivery, there was a severe bleeding complication from the trachea following intubation. The distribution of blood loss is illustrated in Fig 2.

### PPH and subtype of VWD

The median blood loss was higher in women with VWD type 3 compared to the other subtypes, but the difference was not statistically significant (Table 2). The rate of severe PPH differed significantly between the subtypes and was lowest in VWD type 2 (7,1%) and highest in VWD type 3 (75,0%, p = 0,02). The incidence of primary PPH and secondary PPH did not differ significantly between the three subtypes of VWD.

**Table 2:** PPH incidence according to subtype of VWD.

	VWD type 1 (n = 39)	VWD type 2(n = 14)	VWD type 3 (n = 4)	
Median blood loss, ml (range)	450(200–6000)	425(200–1000)	1375(400–3200)	p = 0.63
Primary PPH (>500 ml) %	46.2	35.7	75.0	p = 0.37
Severe primary PPH (>1000 ml) %	20.5	7.1	75.0	**p = 0.02**
Vaginal hematoma	7.7	-	-	p = 0.65
Secondary PPH %	10.3	-	25.0	p = 0.27
Blood transfusion %	7.7	-	-	p = 0.65

A comparison of maternal bleeding complications between the three different subtypes of VWD. P-value calculated using Kruskal-Wallis test for median blood loss and Fisher’s exact test for dichotomous variables.

Abbreviations: PPH = postpartum haemorrhage; VWD = von Willebrand disease; n = number of patients.

**Fig 3:**
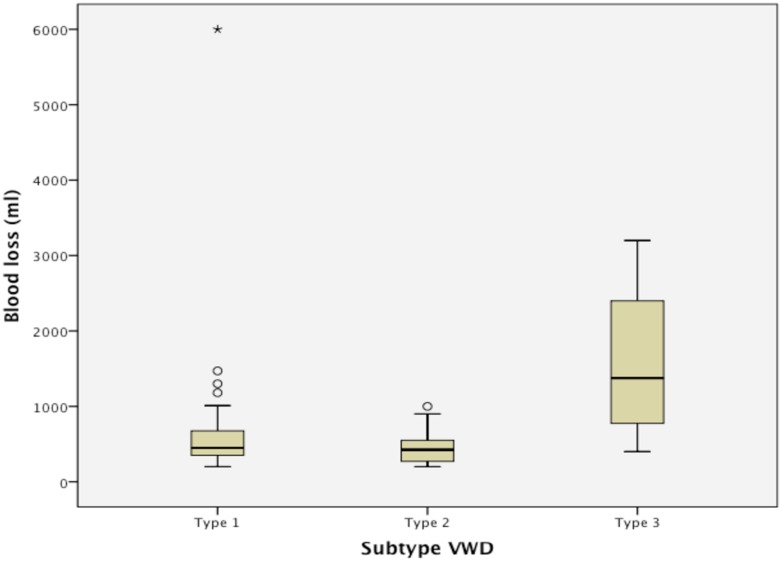
Blood loss (ml) in association with delivery in the different subtypes of von Willebrand disease (VWD).

Apart from one severe case of PPH in VWD type 1, the most severe cases of PPH occurred in VWD type 3 (Fig 3). Two deliveries were excluded from calculation, since the subtype of VWD was unknown at the time of analysis.

### PPH and levels of coagulation factors

There was moderate negative correlation between plasma levels of FVIII:C in third trimester pregnancy and blood loss in association with delivery (r = -0.428, p = 0.01). The levels of VWF activity in late pregnancy did not correlate significantly with blood loss (r = -0.31, p = 0.6).

There was a moderate inverse correlation between the levels of VWF activity in pregnancy and the duration of treatment with CFC postpartum (r = -0.56, p = 0.01), but there was no significant correlation between FVIII activity and treatment duration (r = -0.31, p = 0.17)

### PPH and hemostatic medications

The choice of exact treatment in every case was consistent with published recommendations [31–34].

In all pregnancies where the VWD diagnosis was known before delivery, the patient was prescribed tranexamic acid (TXA, orally or intravenously) every eight hours at the start of labour. Tranexamic acid is an antifibrinolytic agent that acts through blocking lysine sites on plasminogen molecules. Consequently, fibrinolysis is inhibited and excessive bleeding is reduced. During delivery, a sequence of changes occurs that reduce bleeding: myometrial contractions, increased platelet activity, a stormy release of coagulant factors and a parallel increase in the fibrinolytic activity. As a result, there is a rationale for the use of antifibrinolytic agents in the treatment of PPH [35, 36]. The treatment with TXA was continued for a median of 10 days (range 2–14).

In all cases DDAVP or CFC were given on top of TXA. DDAVP usually produces rapid increase in circulating levels of vWF:Ag and FVIII. Thus, DDAVP is considered to be treatment of choice in type 1 VWD patients. However, some patients with type 2 vWD respond well to DDAVP. CFC was used in all patients with type 3 VWD (extremely low levels of vWF) or if single dose of desmopressin failed to provide therapeutic effect. A single dose of desmopressin (DDAVP^®^, Sanofi-Aventis U.S. LLC) was administered in 12 deliveries (11 in type 1 VWD and 1 in type 2 VWD).

**Fig 4:**
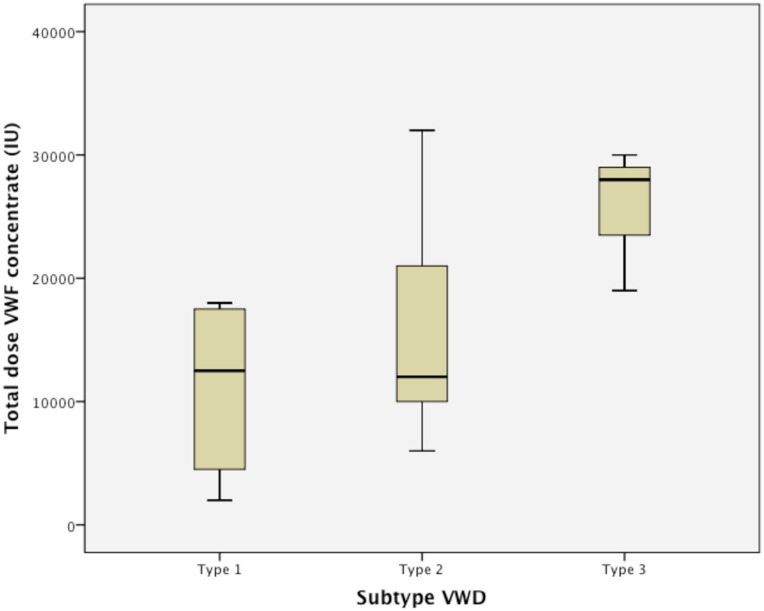
Total dose of clotting factor concentrate (CFC) in the three different subtypes of von Willebrand disease (VWD).

Haemate-P^®^ (CSL Behring GmbH), a clotting factor concentrate (CFC) with plasma-derived VWF and FVIII was given prior to delivery in 22 deliveries. In all but one delivery where CFC was used prophylactically, VWF activity was subnormal (<0.50 kIU/L) during the third trimester. In one case, VWF activity was 0.56 kIU/L. The prophylactic dose of CFC given prior to delivery ranged from 1000–4000 IU (median 2000 IU) and a second dose of Haemate-P^®^ was administered approximately 12 hours later. Haemate-P^®^ was then administered as daily intravenous bolus injections for a median of 9 days (range 1–18). The total amount of CFC given ranged from 2000–35000 IU (Fig 4).

In 16 deliveries among 11 women, the VWD diagnosis was unknown at the time of delivery, and thus the levels of VWF and FVIII had not been analysed during pregnancy. The diagnosis was thus set in a later stage. Consequently, no hemostatic drugs were administered before or after delivery. The majority of these patients had VWD type 1 (81% type 1, 6% type 2, 13% unknown).

The rate of primary PPH (>500 ml) tended to be higher in women when DDAVP or CFC was given compared to when no treatment was given (Table 3). However, the rate of severe primary PPH (>1000 ml) and secondary PPH tended to be lower in all treatment groups compared to when no treatment was given (rates). The sample size was insufficient to show any significant differences in PPH incidence between the different treatment groups.

**Table 3:** PPH incidence and type of haemostatic drug treatment.

	No haemostatic drug treatment (n = 16)	TXA (n = 9)	TXA + DDAVP (n = 12)	TXA + CFC (n = 22)
Median blood loss, ml (range)	425(200–6000)	400(270–1470)	450(200–750)	525(200–3200)
Primary PPH %	46.5	11.1	50	59.1
Severe primary PPH %	31.3	11.1	-	27.3
Vaginal hematoma %	12.5	-	-	4.5
Secondary PPH %	31.3	-	8.3	4.5
Blood transfusion %	18.8	-	-	-

Bleeding complications in association with 59 deliveries in women with VWD depending on treatment regime. Data were insufficient to show statistical significance between the groups.

Abbreviations: VWD = von Willebrand disease; TXA = tranexamic acid; DVAAP = desmopressin; CFC = clotting factor concentrate; PPH = postpartum haemorrhage; n = number of patients.

There was no significant correlation between blood loss and prophylactic dose of CFC (r = 0.03, p = 0.84), nor was there any significant association between secondary PPH and duration of treatment with TXA (p = 0.56), duration of treatment with CFC (p = 0.58) or total dose of CFC (p = 0.64).

### PPH in undiagnosed VWD

The incidence of secondary PPH was significantly higher in women where the VWD diagnosis was unknown at the time of delivery, compared to women where the diagnosis was known (p = 0.013). Primary PPH and severe primary PPH were more common in the women were the diagnosis of VWD was unknown, however difference was not statistically significant. (Table 4).

**Table 4:** PPH incidence in known vs. unknown VWD diagnosis.

	All deliveries (n = 59)	Known VWD diagnosis (n = 43)	Unknown VWD diagnosis (n = 16)	Significance (2-sided)
Median blood loss, ml (range)	450(200–6000)	450(200–3200)	425(200–6000)	
Primary PPH (>500 ml) %	44.1	37.5	46.5	p = 0.57
Severe primary PPH (>1000 ml) %	20.3	16.3	31.3	p = 0.28
Vaginal hematoma	5.1	2.3	12.5	p = 0.18
Secondary PPH %	11.9	4.7	31.3	**p = 0.013**
Blood transfusion %	5.1	-	18.8	**p = 0.017**

A comparison of maternal bleeding complications between deliveries where the patient’s VWD diagnosis was known compared to when the diagnosis was unknown. P-value calculated using Fisher’s exact test.

Abbreviations: PPH = postpartum haemorrhage; VWD = von Willebrand disease; n = number of patients.

Blood transfusion was needed in three cases (18,8%) where the diagnosis of VWD was unknown prior to delivery. Whereas in cases of known diagnosis blood transfusion was not required.

## Discussion

The incidence of PPH was considerably higher in the study group with VWD compared to the general incidence in high resource countries [11, 12, 16, 37]. The incidence of primary PPH was 44% in our study group, thus higher than what has been described in the previous studies where similar methods of data collection were used, and similar to the reported incidence in studies investigating PPH by patient recall [2, 18, 20, 22–24, 38].

In previous studies, the results varied depending on the study design. Studies using interviews and questionnaires for data collection have reported a primary PPH incidence of 31–59% [2, 18, 20, 22, 23], whereas the incidence was 15–34% in studies using an objective measure of blood loss (>500 ml) and medical records for data collection [2, 24, 38, 39]. However, comparing the results between these studies is problematic, as the study groups may differ regarding severity of VWD, hemostatic drug treatment and obstetric care. There are well known difficulties in accurately estimating maternal blood loss, and routines for estimation may differ between hospitals [40].

According to Holmgren [37], the incidence of severe primary PPH in general population is approximately 3.5% for vaginal delivery, 8% for instrumental vaginal delivery and 13% for caesarean section in the general population [37]. In our study group, the incidence of PPH was higher in all delivery modes, and especially high in instrumental vaginal deliveries, where three out of seven deliveries (43%) resulted in a blood loss of more than 1000 ml. Though there is insufficient data to make generalizations, these results strengthen the recommendation that instrumental vaginal delivery should be avoided in women with VWD, in order to prevent genital tract lacerations and severe bleeding episodes [41].

Chee et al. [42] have recently analyzed 61 deliveries among 33 women in Scotland and reported a higher incidence of primary PPH in women where the VWD diagnosis was known and treated, compared to when the diagnosis was unknown. The authors concluded that women with an unknown disease are likely to have milder forms of VWD and therefore be less likely to experience PPH [42]. Our results suggested the opposite with a higher incidence of PPH when the VWD diagnosis was unknown, and thus untreated, even though these patients had milder forms of VWD. One possible explanation of the conflicting results may be differences in prophylactic hemostatic drug treatment. The Nordic Hemophilia Council [43] gives more generous recommendations regarding treatment compared to most other authors. Thus, treatment with TXA is recommended in all deliveries where the patient has VWD, whereas other authors only recommend TXA when the level of VWF activity has been found to be low during the third trimester of pregnancy [43–45]. In the present study, all women with a known VWD diagnosis received TXA prior to delivery.

We were unable to find any significant difference in PPH incidence between women who received TXA and CFC, women who received TXA and DDAVP and women who received only TXA. Since the hemostatic drug treatment varied in our study group, depending on the severity of disease, such differences would be difficult to interpret. Additionally, the subgroups who were administered the different types of treatment were small. The women who received CFC prior to delivery generally had more severe subtypes of VWD and lower levels of VWF and FVIII in pregnancy.

Our results support previous data, saying that low levels of FVIII in the third trimester of pregnancy may predict PPH [38, 46–48], even when CFC prophylaxis is administered, suggesting that the treatment was insufficient in the most severe cases of VWD. Women with type 3 VWD appeared to be at highest risk of severe PPH, even though they all received prophylactic treatment with CFC and TXA prior to delivery. Interestingly, women with VWD type 2 appeared to be less likely to experience severe PPH than women with type 1(p = 0,02). One explanation may be that women with VWD type 1 were overrepresented in the group that did not receive any hemostatic drug treatment. An important finding was that the incidence of primary and secondary PPH was higher than the reported incidence in the general population, even when the diagnosis of VWD was known and managed according to current international guidelines [12–15].

It is worth mentioning that the collected data was in many cases insufficient to make comparisons between subgroups within the material. For example, there were only four deliveries for three women with type 3 VWD. Since VWD is a rare disease, it is difficult to obtain the number of patients required to make such comparisons. To overcome small subgroups problem, data need to be collected in a multi-center study.

The retrospective study itself had some limitations. As previously mentioned, there is a known difficulty in estimating maternal blood loss accurately and the routines may differ between hospitals [40]. The present study included deliveries from 14 different obstetric units over a period of 18 years.

One advantage of the retrospective study design was the inclusion of 11 women who were not diagnosed with VWD at the time of delivery, making comparisons possible between patients who received treatment and patients who did not.

This is to our knowledge the first study investigating the incidence of PPH in women with VWD in Sweden in recent years. All study participants were recruited from the Coagulation Unit at Karolinska University Hospital and their diagnosis of VWD had thus been confirmed according to the recommended diagnostic criteria [7]. The sample size of 59 deliveries in 34 women can be considered as relatively large, as VWD is a rare disorder, based on the referral rate to specialized coagulation centers [8]. Similar sample sizes have been reported in previous studies from England, Scotland and the United States [20, 38, 42].

## Conclusion

Women with VWD in Sweden appear to be at increased risk of experiencing PPH, despite versatile treatment options. That applies especially to women with type 3 VWD or low levels of FVIII activity in late pregnancy and women who gave birth under no hemostatic drug treatment, since the diagnosis of VWD was only established after delivery. It may therefore be of importance to identify women with undiagnosed VWD and previous bleeding history (both personal and family.
