# Correction: Postpartum Hemorrhage in Women with Von Willebrand Disease - A Retrospective Observational Study

**DOI:** 10.1371/journal.pone.0172185

**Published:** 2017-02-09

**Authors:** Igor Govorov, Signe Löfgren, Roza Chaireti, Margareta Holmström, Katarina Bremme, Miriam Mints

The funding statement is incorrect and does not acknowledge a major funder. The correct statement should read: This work was supported with Swedish Institute’s scholarship #13081/2015. The funders had no role in study design, data collection and analysis, decision to publish, or preparation of the manuscript.
